# Development of a Core outcome set for fetal Myelomeningocele (COSMiC): study protocol

**DOI:** 10.1186/s13063-020-04668-6

**Published:** 2020-08-21

**Authors:** Samar Altoukhi, Clare L. Whitehead, Greg Ryan, Jan Deprest, Luc Joyeux, Katie Gallagher, James Drake, Paige Church, Daphne Horn, Yenge Diambomba, Jose C. A. Carvalho, Tim Van Mieghem

**Affiliations:** 1 Department of Obstetrics and Gynaecology, Mount Sinai Hospital and University of Toronto, 600 University Avenue, Toronto, Canada; 2 Department of Obstetrics and Gynaecology, King Fahad Medical City, Riyadh, Saudi Arabia; 3 Department of Obstetrics & Gynaecology, University of Adelaide, Adelaide, Australia; 4 Department of Obstetrics & Gynaecology, University of Melbourne, Melbourne, Australia; 5 Ontario Fetal Centre, Mount Sinai Hospital, Toronto, Canada; 6 Department of Obstetrics and Gynaecology, University Hospitals Leuven, Leuven, Belgium; 7 Institute of Obstetrics and Gynaecology, Women’s Health, University College London, London, UK; 8 Institute of Child Health, University College London, London, UK; 9 Department of Neurosurgery, Hospital for Sick Children and University of Toronto, Toronto, Canada; 10 Department of Neonatology, Holland-Bloorview, Sunnybrook Health Centre and University of Toronto, Toronto, Canada; 11 Department of Medical Informatics, Mount Sinai Hospital and University of Toronto, Toronto, Canada; 12 Department of Neonatology, Mount Sinai Hospital and University of Toronto, Toronto, Canada; 13 Department of Anaesthesia, Mount Sinai Hospital and University of Toronto, Toronto, Canada

**Keywords:** Spina bifida, Myelomeningocele, Myeloschisis, Fetal surgery, Fetoscopic surgery, Core outcome set, Delphi study, Open spina bifida

## Abstract

**Background:**

Open spina bifida (OSB) is one of the most common congenital central nervous system defects and leads to long-term physical and cognitive disabilities. Open fetal surgery for OSB improves neurological outcomes and reduces the need for ventriculoperitoneal shunting, compared to postnatal surgery, but is associated with a significant risk of prematurity and maternal morbidity. Fetoscopic surgery comes with less maternal morbidity, yet the question remains whether the procedure is neuroprotective and reduces prematurity. Comparison of outcomes between different treatment options is challenging due to inconsistent outcome reporting. We aim to develop and disseminate a core outcome set (COS) for fetal OSB, to ensure that outcomes relevant to all stakeholders are collected and reported in a standardised fashion in future studies.

**Methods:**

The COS will be developed using a validated Delphi methodology. A systematic literature review will be performed to identify outcomes previously reported for prenatally diagnosed OSB. We will assess maternal (primary and subsequent pregnancies), fetal, neonatal and childhood outcomes until adolescence. In a second phase, we will conduct semi-structured interviews with stakeholders, to ensure representation of additional relevant outcomes that may not have been reported in the literature. We will include patients and parents, as well as health professionals involved in the care of these pregnancies and children (fetal medicine specialists, fetal surgeons, neonatologists/paediatricians and allied health). Subsequently, an international group of key stakeholders will rate the importance of the identified outcomes using three sequential online rounds of a modified Delphi Survey. Final agreement on outcomes to be included in the COS, their definition and measurement will be achieved through a face-to-face consensus meeting with all stakeholder groups. Dissemination of the final COS will be ensured through different media and relevant societies.

**Discussion:**

Development and implementation of a COS for fetal OSB will ensure consistent outcome reporting in future clinical trials, systematic reviews and clinical practice guidelines. This will lead to higher quality research, better evidence-based clinical practice and ultimately improved maternal, fetal and long-term childhood outcomes.

**Trial registration:**

International Prospective Register of Systematic Reviews (PROSPERO) CRD42018104880. Registered on December 5, 2018. Core Outcome Measures in Effectiveness Trials (COMET): 1187

## Background

Open spina bifida (OSB) is a common birth defect, complicating approximately 5 in every 10,000 births [1–3]. The condition occurs when the lower segments of the neural tube do not close properly early in embryonic life. As a result, the fetal meninges and the nerve elements are exposed at the level of the fetal back. The condition is not lethal and, with access to multidisciplinary prenatal and postnatal care, 1-year survival rates are 88–96% [2, 4]. However, progressive damage to the exposed neural elements in utero results in distal spinal cord and nerve dysfunction. Moreover, progressive leakage of cerebrospinal fluid through the defect causes hindbrain herniation (Chiari 2 malformation) and ventriculomegaly/hydrocephalus [5]. Clinically, this presents as impaired lower limb function (ambulation problems), bowel and bladder dysfunction (constipation and incontinence) and sexual dysfunction postnatally. About 15% of cases will have decreased cognitive function, in part due to complications of ventriculo-peritoneal shunt placement, which is often required postnatally to relieve the increased intra-cranial pressure resulting from the hydrocephalus [3, 6].

Historically, the spinal lesion was closed surgically shortly after birth (postnatal repair), but fetal repair is now becoming the state-of-the-art treatment as it improves functional outcomes [7–11]. Indeed, in 2011, the Management of Myelomeningocele Study (MOMS), a randomised controlled trial comparing prenatal to postnatal repair, demonstrated that in-utero closure resulted in a reversal of hindbrain herniation in two thirds of cases, halved the need for ventriculoperitoneal shunting and improved motor function. However, fetal surgery comes at the cost of an increased risk of maternal morbidity, preterm rupture of membranes and preterm delivery [12].

The MOMS trial utilised a hysterotomy-based approach for prenatal repair, also referred to as ‘open’ fetal surgery, but subsequent minimally invasive (fetoscopic) approaches have been developed in an attempt to minimise fetal and maternal morbidity [13, 14]. Given the rapid developments in prenatal interventions, there is a need for robust evidence on the safety and efficacy of these therapies [15]. The literature to date reports varying outcomes, which makes it hard to compare results or synthesise the data from multiple studies, thereby limiting the value of this research in providing guidance to clinical practice.

Core outcome sets (COS) are well-defined, disease-specific, standardised groups of outcome measures that should be recorded and reported in all trials [16]. The development of COS is supported by the Core Outcome Measures in Effectiveness Trials initiative (COMET) (www.comet-initiative.org) and further promoted in women’s health by the Core Outcomes in Women’s and Newborn’s Health (CROWN) initiative [17]. COSs in women’s health have now been established for many disease conditions, with demonstrable clinical benefits [18].

Our goal is to develop, disseminate, and implement a core outcome set for prenatally diagnosed spina bifida that incorporates the views of key stakeholders including health professionals, researchers, parents and children.

## Methods

### Overview

**Fig. 1:**
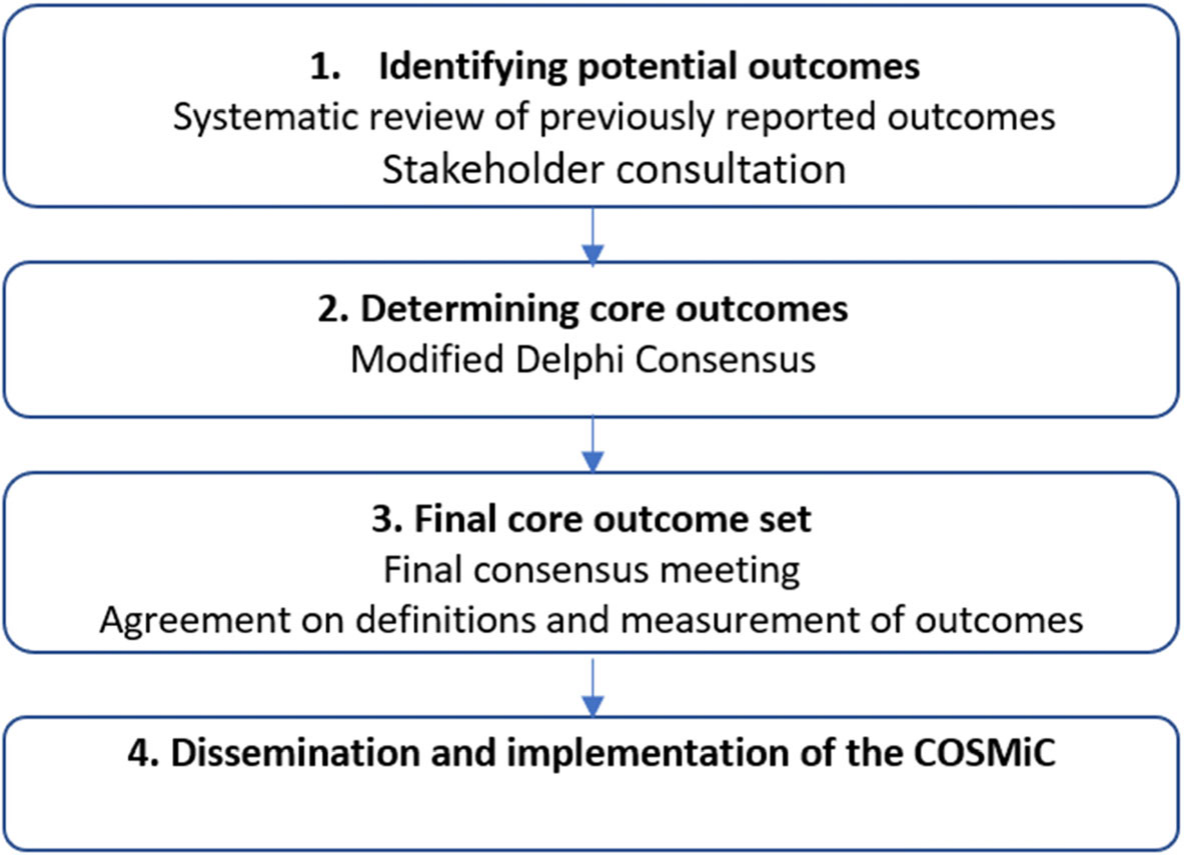
Stages for developing a Core Outcome Set for prenatally diagnosed Myelomeningocele (COSMiC)

The methods for the development of the Core Outcome Set for Myelomeningocele (COSMiC) are informed by the recommendations of the COMET initiative and the Core Outcome Set–STAndards (COS-STAD), with adaptations specific to the scope of this project [19, 20]. We will use a step-wise approach, consisting of four stages, as shown in Fig. 1. Step 1 will be to identify currently reported outcomes in the literature and identify unreported outcomes important to patient/parent stakeholders, through structured interviews. Step 2 will be a modified three-phase online Delphi process to determine the core outcomes important to all stakeholders. Step 3 will be a face-to-face consensus meeting of key stakeholders to agree on the COS, and step 4 will be dissemination and implementation of the developed COS.

### Study registration

The study has been registered with the COMET initiative (www.comet-initiative.org; # 1187) and the International Prospective Register of Systematic Reviews (PROSPERO; # CRD42018104880). The systematic review will be conducted in accordance with guidance set out by the PRISMA Statement for reporting systematic reviews and meta-analyses of studies that evaluate health care interventions.

### Scope of the core outcome set

This core outcome set will apply to all pre- and perinatal interventions for prenatally diagnosed OSB. We will include outcome measures related to maternal, fetal, neonatal and childhood outcomes.

### Systematic review to identify potential core outcomes

We will conduct and publish a systematic review to identify what outcomes have previously been reported for prenatally diagnosed OSB. Studies reporting on fetal surgery, termination of pregnancy and/or postnatal repair will be included. We will include randomised controlled trials and observational retrospective and prospective studies. We will exclude case reports, case series including less than 20 cases, editorials, letters to the editor and review articles. All outcomes reported in the studies will be considered.

Our search strategy was designed by an experienced librarian (DH) and contains variations on the following MeSH terms: Meningomyelocele, myelocele, myelomeningocele, Spinal dysraphism, spina bifida cystica, meningocele, fetus, pregnancy, in-utero, intra-uterine, antenatal, prenatal, fetal diseases, fetal therapies and prenatal diagnosis. We will search the databases EMBASE, MEDLINE, CINAHL, PubMed and ClinicalTrials.gov from inception to September 2019. No data or language limits will be applied. Two reviewers (SA and CW) will individually screen all titles and abstracts of identified manuscripts. The full-text articles will be reviewed for all studies that meet the inclusion criteria, in addition to the studies where inclusion cannot be decided based solely on the abstract or title. The articles will be assessed for eligibility based on a critical review of the studies’ full text and any conflicts between the reviewers will be resolved through dialogue and arbitration by a third reviewer (TVM), should this be necessary. Reference management will be done with DistillerSR (Evidence Partners, Ottawa, Canada).

Data will be extracted using a predesigned and trialled proforma. The following information will be included for each study: study design, year of study, journal, sample size, setting, participants, interventions (if any), outcomes, outcome measurement tools and timing of outcome measurement.

All identified outcomes will be collated into an outcome inventory and organised into the following categories: maternal (primary and subsequent pregnancies), fetal, neonatal and childhood to adolescence. The outcomes will be reviewed by the steering committee to reduce duplication and ensure the final inventory is succinct and clear. The steering group will consist of fetal medicine specialists, fetal surgeons (obstetricians, neurosurgeons and paediatric surgeons), neonatologists and developmental paediatricians, allied health specialists, as well as a group of patient participants. The inventory will be entered into the modified Delphi process.

### Patient and stakeholder involvement

Patient, parent and carer expertise is crucial for the development of COSs. Patient participants frequently identify outcomes that may not be considered clinically relevant by health professionals or not yet recognised in the existing literature. Semi-structured qualitative interviews will be performed with patients, parents and carers affected by prenatally diagnosed OSB to identify patient reported outcomes and outcome domains [19]. A minimum of two groups of parents will be included: those who underwent prenatal OSB repair and those who continue the pregnancy with a postnatal repair. Interviews will be audio-recorded, transcribed and analysed for content. Patient participants will also be asked to assess the words, phrases and language used to define and describe conditions, interventions and outcomes to be included in the Delphi process. Similar semi-structured interviews will also be held with other aforementioned stakeholders.

### Determining core outcomes

The core outcomes will be determined using a modified Delphi technique [21]. Electronic questionnaires will be used to facilitate international consensus building. We will include Delphi participants across all involved stakeholder groups. Potential experts will be identified by members of the steering committee who represent a diverse and international field of expertise. Experts from established and experienced centres that perform both prenatal and postnatal repair and regularly publish their results, as well as those where only postnatal repair is offered, will be invited to participate. Additional invitations will be extended to national and international professional organisations to advertise the study and encourage additional participation, as well as through social media. International coverage will be prioritised and will be facilitated by the international representation of the steering group (North America, Europe, Middle East, Asia/Pacific). Patient participants will be invited from fetal and paediatric centres specialising in prenatal diagnosis and treatment of OSB, as well as through patient support groups.

Participants will be sent a plain language information summary of the study, explanation of the Delphi process and further instructions on how to participate. Participants will be asked to forward the invitation to other potential participants with expertise in the field. Once the participants register and consent to participate, they will complete an online demographic survey and be asked to self-identify with the most appropriate stakeholder group. Participants who fail to complete all three rounds will be asked for their reason for withdrawal.

### Delphi process

There will be three online sequential rounds of the Delphi process. Questionnaires will be completed using DelphiManager, a web-based system for management of Delphi surveys (COMET Initiative, University of Liverpool, UK). Each participant will be given a unique identifier that will be used throughout the process and ensure anonymity is maintained. In each round, participants will be sent a link to the questionnaire and their responses collated and analysed for future rounds.

#### First round

The first round will ask participants to rate each of the outcomes using a 9-point Likert scale where typically 1–3 signifies an outcome that is of minor importance or relevance, 4–6 is important but not critical, while 7–9 signified a critical outcome [22]. We will also include an ‘unable-to-score’ category for those participants who feel they may not have the expertise to assess certain outcomes. The outcomes will be grouped under relevant headings and categories (maternal, fetal, neonatal, childhood). Participants in this round will also be encouraged to include ‘new’ outcomes that they would consider relevant for inclusion but which have not previously been identified. Explanations of the outcomes in lay terms will be included where necessary. The survey will remain open for 6 weeks with biweekly reminders to encourage completion of the survey. At the end of the round, individual answers will be aggregated and summarised. New outcomes will be considered and potentially approved by the Steering Group for inclusion in the second round.

#### Second round

Only participants who completed round one will be invited to complete round two. All outcomes from round one in addition to the new approved outcomes provided by the participants will be included in the second round. Each participant will receive the rating of each outcome for individual responses, as well as summative responses for each stakeholder group and the total group overall.

Participants will be asked to re-rate the outcomes using the same Likert scale as in round one, with the knowledge of their previous response and with the knowledge of other stakeholders’ ratings. Participants may change their ratings if they consider that rescoring is more appropriate or retain their original score.

Prior to submission of the round two responses, participants will be asked if they are interested in participating in the face-to-face consensus meeting after round three.

#### Third round

Participants who completed round two will be presented with the results of the previous round at the individual, stakeholder and overall group level and asked to accept or reject those were consensus was reached. We are defining ‘consensus’ as an outcome that at least 70% of the panel members have rated as critically important (7–9) and less than 15% have rated as not important (1–3) [23]. Outcomes will additionally be classified as ‘consensus out’ if > 70% participants rated it 1–3 and < 15% rated it 7–9. Outcomes which fulfil these criteria at the conclusion of the final round will move on to the consensus meeting. There is currently no universally accepted definition of consensus as to which scores on the 9-point scale indicate items that should be brought to a COS consensus meeting, but recent COSs, including in women’s health research, have used similar definitions [24, 25]. Willingness to participate in the face-to-face meeting will be confirmed.

### Stakeholder consensus

Participants who complete all three phases of the Delphi process and express an interest to attend will be invited to the consensus meeting. Our aim is to involve 2–5 representatives from each of the stakeholder groups. Should important stakeholders be unable to attend in person, electronic meeting software will be used. Discussions will be led by an experienced moderator to ensure the meeting is collaborative, inclusive and productive. The main objective of the meeting will be to approve the final COS from the Delphi process and address outcomes where consensus was not previously reached. Each of the outcomes not reaching consensus will be discussed and a final vote on their inclusion/exclusion performed anonymously and electronically.

### Measuring core outcomes

Once the final COS has been agreed upon, we will reach consensus on how and when the outcomes should be measured. Potential outcome definitions will then be discussed and agreed upon at the consensus meeting based on data from our systematic review. Measurement methods will be assessed for quality based on the framework of the Consensus-based Standards for the selection of health Measurement Instruments (*www.cosmin.nl*). COSMIN evaluates outcome measures according to their validity, reliability, responsiveness and interpretability [26]. If the final outcomes were not identified in our systematic review, we will conduct an additional electronic search exercise to determine the most appropriate measurement tool(s).

### Dissemination and implementation

We will collaborate with researchers and journal editors from relevant maternal-fetal medicine, surgical and paediatric fields to distribute and implement the COS as widely as possible. We plan on presenting the COS at national and international meetings and publishing in peer reviewed journals endorsed by the CROWN initiative and relevant to the key stakeholder groups. The CROWN initiative and Cochrane Pregnancy and Childbirth Group endorse COS and expect future authors to report their study results according to the COS, rather than non-core or surrogate outcomes [17]. We will also disseminate it further through press-releases, newsletters and events to raise awareness of its development especially amongst parent/patient organisations. We will engage with Cochrane Review Groups, clinical guideline developers, research funders and regulators and trial registries to support its implementation. Our protocol and its results will be archived in COMET and CROWN databases to aid ease of accessibility for future studies on prenatally diagnosed OSB [26].

## Discussion

Rapid advances in prenatal diagnosis and fetal surgery highlight the need for the development and implementation of a standardised core outcome set for prenatally diagnosed OSB. This COS will be vital to guide the design of future clinical trials and reporting on novel surgical techniques. Involvement of parents and patients affected by prenatally diagnosed OSB will ensure that the COS will be clinically relevant and wide-reaching. It will allow the effective standardisation and harmonisation of data for systematic reviews and meta-analyses and ultimately improve clinical practice and patient care. We hope that the principles of this COS may also guide further research in other areas of fetal surgery that are currently being developed.

## Trial status

The systematic review part of this study has been registered with the International prospective register of systematic reviews (Prospero) on December 5, 2018, with study number CRD42018104880 and started on January 1, 2019. Data collection is complete and analysis is ongoing. Stakeholder interviews will start in the fall of 2020. The Delphi process will follow, with completion expected by June 2021.

This is the first version of the protocol.

## Data Availability

Not applicable

## Abbreviations

COS: Core outcome set
OSB: Open spina bifida
COMET: Core Outcome Measures in Effectiveness Trials
CROWN: Core Outcomes in Women’s and Newborn’s Health
COS-STAD: Core Outcome Set–STAndards
COSMIN: Consensus-based Standards for the selection of health Measurement INstruments
CENTRAL: Cochrane Central Register of Controlled trials
GRADE: Grading of Recommendations Assessment, Development and Evaluation
PRISMA: Preferred Reporting for Systematic Reviews and Meta-Analyses
MOMS: Management of Myelomeningocele Study
